# Biased dispersal can explain fast human range expansions

**DOI:** 10.1038/s41598-020-66045-2

**Published:** 2020-06-03

**Authors:** Joaquim Fort

**Affiliations:** 1 Complex Systems Laboratory, Universitat de Girona, C/ Maria Aurèlia Capmany 61, 17003 Girona, Catalonia Spain; 2 Catalan Institution for Research and Advanced Studies (ICREA), Passeig Lluís Companys 3, 08010 Barcelona, Catalonia Spain

**Keywords:** Nonlinear phenomena, Dynamical systems

## Abstract

Some human fronts spread faster than expected by models based on dispersal and reproduction. The only explanation proposed so far assumes that some autochthonous individuals are incorporated by the expanding populations, leading to faster front speeds. Here we show that simple models without this effect are also consistent with the observed speeds of two fronts (a Khoi-khoi expansion of herders and a Bantu expansion of farmers), provided that the dispersal of individuals is biased (i.e., more probable) in directions closer to the front propagation direction. The physical models presented may also be applied to other kinds of social phenomena, including innovation diffusion, rumor propagation, linguistic fronts, epidemic spread, diffusion in economic space and the evolution of cooperation in spatial systems. They can be also adapted to non-human systems with biased dispersal, including biological invasions, cancer tumors and virus treatment of tumors.

## Introduction

It is reasonable to expect that some processes involving human populations may be modelled using equations of statistical Physics^1^. One way to see this is by noting that, although different individuals may take different decisions in the same situation, the dynamics of populations composed of many individuals can be related to the probabilities that an arbitrary individual takes different decisions. A well-known example is the propagation of human population fronts (also called waves of advance)^2,3^, first introduced by Fisher^4^ and Kolmogorov *et al*.^5^ in a genetic context and later applied by Ammerman and Cavalli-Sforza to the spread of Neolithic populations in Europe^6,7^. Here we will also deal with some Neolithic fronts, but our methodology can be applied to historical case studies in which a population increases its number and expands it range during generations, e.g., the colonization of the USA in the 19th. century^8^.

One reason for the wide interdisciplinary interest in the Neolithic transition is that it is considered the most important shift in human prehistory and history, because it replaced hunting-gathering by agriculture, which opened new possibilities to store food and eventually led to urbanization, an increasing division of labor, writing, mathematics, etc. (for example, most hunter-gatherer societies do not have numbers larger than five^9^). Present human societies are based on these cultural innovations, but none of the latter were introduced within the millions of years during which humans lived as hunter-gatherers (indeed, all of those innovations appeared only within the last 12,000 years). It thus seems impossible to understand the origins of modern society without considering the Neolithic transition. A second reason is that Neolithic and other major prehistoric migrations have shaped the present distributions of genes^10^ and languages^11^. A third reason is that the individuals of prehistoric societies usually had more limited mobility than at present, because modern means of transportation (trains, cars, airplanes, etc.) were absent. This presumably makes prehistoric societies useful as a base case study that allows to describe mathematically several important issues that should be understood before attempting more complicated frameworks. However, understanding prehistoric migrations is not straightforward^12^ (as we shall see below) and there are exceptions even to the intuitively very reasonable expectation of limited mobility in prehistory (for example, long sea travels seem necessary to understand the spread of the Neolithic in the western Mediterranean^13,14^ and Oceania^15^).

Human range expansions (both in prehistory and history) are only a small subset of spread phenomena in human populations that follow similar dynamics, such as infections, languages, and many cultural traits. Early physical models of human range expansions^6,7^, based on reaction-diffusion equations^4,5^, were later generalized to take into account two issues. First, the so-called cohabitation effect, i.e. the fact that newborn humans have to spend some time with their parents before they can separate from them and survive on their own^3,16^. Second, the finding that the diffusive approximation (i.e., describing dispersal by means of diffusion coefficients) can lead to substantial errors compared to using the complete probability of human dispersal as a function of distance (which is called the dispersal kernel)^17^. Since both effects introduce important corrections, they will be included below (mathematical details will be given in the next section).

The work reported in the present paper is motivated by the recent finding that two different human fronts^18,19^ display faster spread rates than expected by the models summarized above. Those models are based only on human dispersal and reproduction, i.e. on the so-called demic or demographic diffusion^7^. The populations involved in these two studies are the Khoi-khoi that spread from about 2,300 until 1,100 yr Before Present (BP) and replaced hunting-gathering by herding in southwestern Africa (with a front speed of 1.4–3.3 km/yr)^18^ and the Bantu farmers that spread southwards from the Great Lakes area from about 3,400 until 1,400 yr BP and replaced hunting-gathering by farming in southeastern Africa (with a front speed of 1.5–2.3 km/yr)^19^. These speed ranges were estimated by linear regressions of archaeological dates and distances^18,19^. The only explanation proposed so far (for the fact that these speeds are higher than those predicted by demic diffusion models) is based on a physical model derived some years ago using cultural transmission theory^12^, according to which the conversion of hunter-gatherers into herders or farmers (i.e., cultural transmission^7^) can lead to substantially faster speeds than purely demic models^18,19^. However, here we will show that simple models without cultural transmission are also consistent with the observed front speeds, provided that the dispersal of individuals is biased (i.e., more probable) in directions closer to the front propagation direction. This seems a reasonable alternative to the usual assumption of isotropic dispersal^6,7,16,17^, because humans will presumably tend to migrate, on average, to locations with more opportunities to develop their subsistence economy (i.e., places still empty of the population that expands its range). We would like to stress, however, that analytical models such as that due to Fisher, Kolmogorov *et al*.^4,5^ and those introduced below are necessarily less detailed that numerical simulations, in the sense that some factors that can affect the front speed are not included, e.g., the irregularity of resources, slowdowns or accelerations due to non-homogenous altitude, climate, density of hunter-gatherers, etc. Moreover, individual displacements into new areas are usually preceded by exploratory movements, and consequently the worst locations may remain empty for a long time after the passage of the front. In future work it could be of interest to develop models that incorporate such factors^20^ and/or describe the evolution behind the front^21^, but here we only look for the simplest possible models able to describe its overall dynamics (i.e., its average speed, which is often the only statistically sound estimation available from the data available).

Although we focus on prehistoric migrations, our results may be also of interest in other social applications of statistical physics including innovation diffusion^22^, rumor propagation^23^, linguistic fronts^24^, epidemic spread^25^, diffusion in economic space^26^ and the evolution of cooperation in spatial systems^27^. The theory developed below can be also modified for application to ecology (biological invasions^28^), microbiology (growth of bacterial colonies and cancer tumors^29^), medicine (virus treatment of tumors^30^), etc. We shall give some details on these additional possible applications after deriving our new results and analyzing their implications on the two Neolithic fronts mentioned above.

## Biased dispersal in human population fronts

Fisher^4^ and Kolmogorov *et al*.^5^ simultaneously derived the following reaction-diffusion equation

1 $$\frac{\partial N}{\partial t}=D\,{\nabla }^{2}N+F(N),$$

where *t* is the time, $N$ the population density (of Neolithic individuals in our case), $D$ its diffusion coefficient and $F(N)$ its growth function, i.e. the net reproduction (births minus deaths per unit time). Usually a logistic function is used for $F(N)$ because it is well-known to agree with many human population data (see^16^ and references therein), i.e.,

2 $$F(N)=aN\left(1-\frac{N}{K}\right),$$

where $a$ is the initial growth rate and $K$ the carrying capacity (maximum population density). Fisher’s Eq. (1) can be obtained by performing Taylor expansions in the so-called non-cohabitation equation [Eq. (3) below], which is derived by applying simply that the variation in population density at position $(x,y)$ is due to individuals arriving minus those leaving plus the net reproduction, i.e^16^.

3 $$\begin{array}{rcl}N(x,y,t+T)-N(x,y,t) & = & {\int }_{-\infty }^{\infty }{\int }_{-\infty }^{\infty }N(x-{\Delta }_{x},y-{\Delta }_{y},t)\,\phi ({\Delta }_{x},{\Delta }_{y})d{\Delta }_{x}d{\Delta }_{y}\\  &  & -N(x,y,t)+{R}_{T}[N(x,y,t)]-N(x,y,t),\end{array}$$

where $T$ is the generation time (mean time interval between the migration of a parent and one of her/his children)^16,31^. The dispersal kernel $\phi ({\Delta }_{x},{\Delta }_{y})\,$can be defined so that $\phi ({\Delta }_{x},{\Delta }_{y})d{\Delta }_{x}d{\Delta }_{y}$ is the probability that an individual born at $(x-{\Delta }_{x},y-{\Delta }_{y})$ and time $t\,$will have a child born between (*x*, *y*) and $(x+d{\Delta }_{x},y+d{\Delta }_{y})$ at time $t+T$ (i.e., that a jump in the random walk will have *x-*coordinate between ${\Delta }_{x}$ and ${\Delta }_{x}+d{\Delta }_{x}$and *y*-coordinate between ${\Delta }_{y}$ and ${\Delta }_{y}+d{\Delta }_{y}$)^16^. Also in Eq. (3), ${R}_{T}[N(x,y,t)]$ is the effect of logistic net reproduction, i.e^17,32^.

4 $${R}_{T}[N(x,y,t)]=\frac{{e}^{aT}K\,N(x,y,t)}{K+({e}^{aT}-1)\,N(x,y,t)}.$$

Note that Eqs. (3) and (4) without dispersal yield the same result as integrating the logistic differential equation[Equations (1)-(2), also without diffusion], between times $t$ and $t+T$. In the limit $T\to 0$, Eq. (4) tends to $N(x,y,t)$, and so does the first term on the right-hand side of Eq. (3) because dispersal distances tend to zero, i.e., $\phi ({\Delta }_{x},{\Delta }_{y})$ tends to a Dirac delta centered at $({\Delta }_{x},{\Delta }_{y})=(0,0)$.

By performing Taylor expansions up to second order in space and first order in time in the non-cohabitation Eq. (3) and assuming an isotropic kernel, it is easy to obtain the Fisher Eq. (1)^16^, which was applied by Ammerman and Cavalli-Sforza to model the spread of the Neolithic in Europe^6,7^. However, in contrast to many physical and chemical systems, it has been shown that for human populations the speed of front solutions to Fisher’s Eq. (1) is not a valid approximation to the more precise Eq. (3)^17^. Moreover, newborn humans cannot survive separated from their parents during some years and for this reason, the non-cohabitation Eq. (3) should be replaced by the so-called cohabitation equation, namely^17,33,34^

5 $$N(x,y,t+T)={R}_{T}[{\int }_{-\infty }^{\infty }{\int }_{-\infty }^{\infty }N(x-{\Delta }_{x},y-{\Delta }_{y},t)\phi ({\Delta }_{x},{\Delta }_{y})d{\Delta }_{x}d{\Delta }_{y}].$$

**Figure 1:**
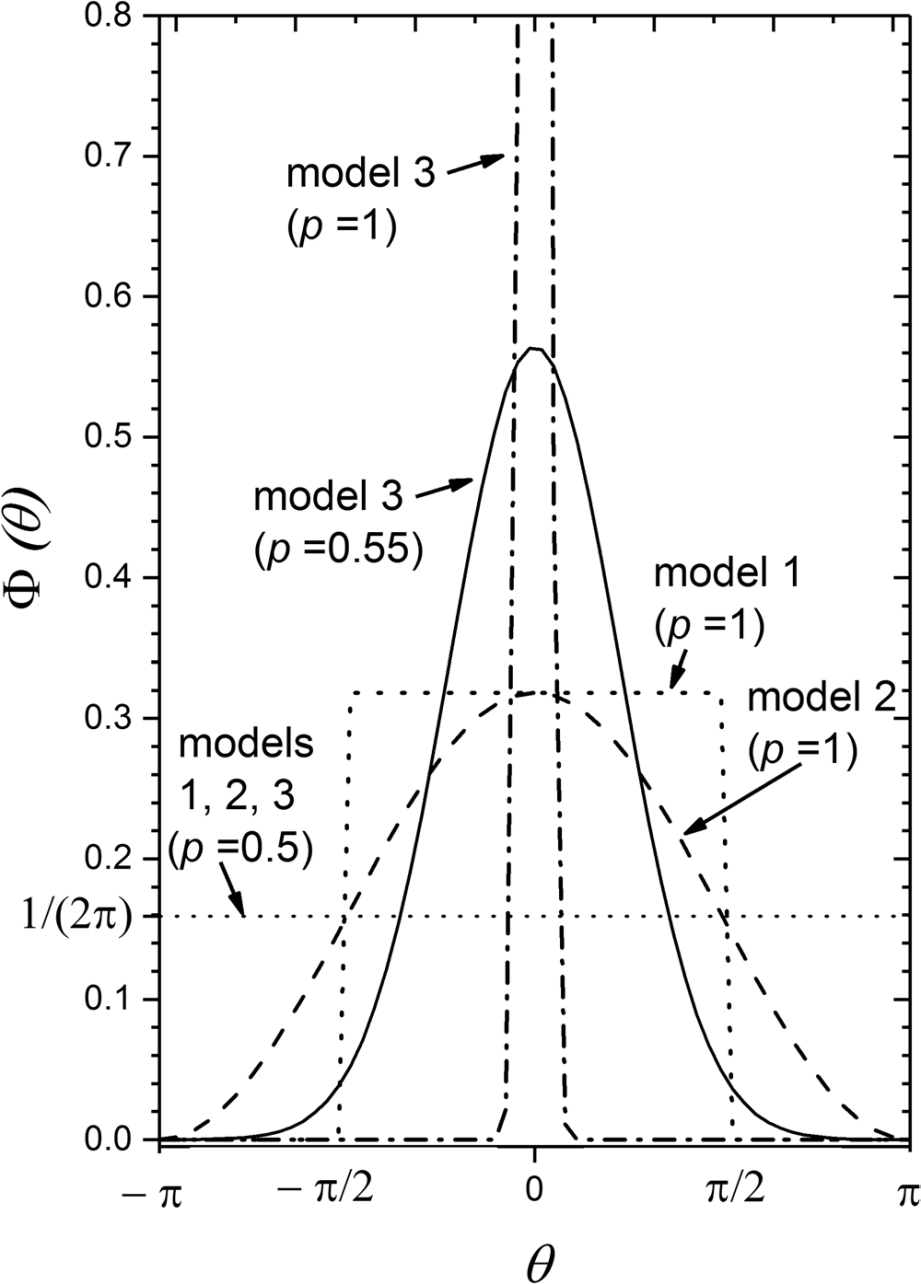
This figure shows some examples of the three models used in this paper. The probability of dispersal $\Phi (\theta )$ is a function of the angle $\theta$ between the direction of migration of an individual and that of the front propagation. In model 1 the distribution $\Phi (\theta )$ is given by Eq. (11), in model 2 it is given by Eq. (13), and in model 3 by Eq. (15). For all three models, the bias parameter $p$ has the value $p=0.5$ if the distribution is isotropic (i.e., non-biased), as shown by the dotted horizontal curve. For the mathematically simpler models 1 and 2 the distributions for the maximum possible value of the bias parameter ($p=1$) are shown as short-dashed and dashed curves. Still more biased dispersal is possible in model 3, for which two biased examples are also shown ($p=0.55$ and $p=1$). For model 3, the bias parameter $p$ has no upper limit and the case $p\to \infty$ corresponds to a Dirac delta centered at $\theta =0$, i.e., all individuals moving in the front propagation direction.

A simple way to see that the cohabitation Eq. (5) is more appropriate for humans that the non-cohabitation Eq. (3) is to note that if there are individuals only at $(x,y,t)$, according to the last two terms of Eq. (3) all newborn children will appear at $(x,y,t+T)$ but the first term on the right of Eq. (3) implies that parents that have migrated away from $(x,y)$ will have left their children there (see Fig. 1 in^17^). This is appropriate for some biological species but not for humans^33^. In contrast, according to Eq. (5) parents and children will be at the same final position. It is important to note that in the cohabitation Eq. (5) we have assumed that parents reproduce after migrating, but the speed of front solutions is the same if they migrate after reproducing and even if they reproduce during migration (for a detailed discussion of this point, see ref. and^33^ and Fig. 17 in ref. ^3^). It is well-established that for human populations it is necessary to use Eq. (5) rather than (3) or (1)^3,17,33^, so the rest of this paper is based on the cohabitation Eq. (5).

As shown in previous work^17,33^, the speed of front solutions to Eq. (5) can be derived by considering the leading edge of the front, i.e., assuming that $N(x,y,t) < K$ in Eq. (4), choosing the positive $x$-axis along the local front propagation direction and using the ansatz $N(x,y,t)\approx {N}_{0}{e}^{-\lambda (x-ct)}$ in Eq. (5), where $\lambda  > 0$ and $c$ is the front speed. Then^17,33^

6 $${e}^{\lambda cT}={e}^{aT}{\int }_{0}^{\infty }\Delta {\rm{d}}\Delta \Psi (\Delta ){\int }_{-\pi }^{\pi }d\theta \,{e}^{\lambda \varDelta \cos \theta }\,\Phi (\theta ),$$

where $\theta =\arccos \frac{{\Delta }_{x}}{\Delta }$ is the angle between the individual displacement $({\Delta }_{x},{\Delta }_{y})$ and the front propagation direction ($x > 0$). For later use, we have assumed that the dispersal kernel can be written as

7 $$\phi ({\Delta }_{x},{\Delta }_{y})=\Psi (\Delta )\Phi (\theta ),$$

where $\Psi (\Delta )\,$is the probability of migration as a function of distance $\Delta$ and $\Phi (\theta )\,$is the probability of migration as a function of direction $\theta$. This is justified if the migration distance $\Delta$ is statistically independent of the migration direction $\theta$.

In recent years, the framework summarized above has been generalized by adding cultural transmission, i.e., an indigenous population (e.g., hunter-gatherers) the individuals of which can join the invading population (e.g., herders or farmers)^12^. In contrast, here we will introduce an alternative model that does not assume cultural transmission but, instead, allows for the possibility that individuals disperse with different probabilities in different directions. This seems reasonable because humans in a population invading a landscape will likely tend to migrate to places where the population density is still low, i.e., the probability of dispersal or migration will increase for directions closer to that of the front propagation (lower values of $\theta$) and this should lead to faster fronts than isotropic (i.e., non-biased) dispersal. It is important to distinguish the effect that we will analyze in this paper (biased dispersal) from a different effect that leads to similar equations and is called correlated dispersal^35–39^. In the latter case individuals also perform a random walk, but the probability of the direction of motion does not depend on its angle relative to a fixed direction (as in biased dispersal) but on the angle relative to the direction of motion during the previous step of the random walk (i.e., during the previous generation in human expansions). We do not deal with this case (correlated dispersal) because we see no reason to think that the direction between, e.g., the birthplace of an individual (A) and one of his children can be related to the direction between the birthplace of the first individual (A) and that of his mother or father. For a detailed comparison between biased and correlated random walks, see ref. ^37^.

As in previous work^2,7,12,18,19^, we can use ethnographic data from preindustrial populations for the probability $\Psi (\Delta )\,$of migration as a function of distance $\Delta$. Such data are usually reported as histograms, i.e., a set of distances and the corresponding probabilities,

8 $$\Psi (\Delta )=\mathop{\sum }\limits_{i=1}^{n}{p}_{i}\frac{{\delta }^{(1)}(\Delta -{r}_{i})}{\Delta },$$

where ${r}_{i}$ is the mean distance of the *i*-th bin in the histogram, ${p}_{i}$ is its probability ($\mathop{\sum }\limits_{i=1}^{n}{p}_{i}=1$) and ${\delta }^{(j)}(\Delta -{r}_{i})$ is the 1-dimensional Dirac delta centered at $\Delta ={r}_{i}$. A simple way to derive the right-hand side of Eq. (8) is to consider Eq. (7) and the normalization conditions

9 $$1={\int }_{-\infty }^{\infty }{\int }_{-\infty }^{\infty }\phi ({\Delta }_{x},{\Delta }_{y})d{\Delta }_{x}d{\Delta }_{y}={\int }_{0}^{\infty }\Delta {\rm{d}}\Delta \Psi (\Delta ){\int }_{-\pi }^{\pi }d\theta \,\Phi (\theta )$$

and

10 $$1={\int }_{-\pi }^{\pi }d\theta \,\Phi (\theta ).$$

Unfortunately, in contrast to $\Psi (\Delta )$, for preindustrial populations there are no data at present that allow to measure the probability $\Phi (\theta )$ of migration as a function of the angle $\theta$ between the direction of individual dispersal $({\Delta }_{x},{\Delta }_{y})$ and that of the front propagation (although such data will hopefully become available in the following years, as explained in the Conclusions section below). Therefore we will introduce and compare three increasingly complex models of biased (or non-isotropic) dispersal, i.e., three different functions $\Phi (\theta )$. This will be enough to fulfill our aim, namely to determine whether or not biased dispersal is a viable mechanism to explain the fastness of the Neolithic fronts of herders^18^ and famers^19^ mentioned above. Intuitively we expect that $\Phi (-\theta )=\Phi (\theta )$, i.e. that dispersal depends only on the magnitude of the angle $\theta$ between the dispersal jump and the front propagation direction ($\theta =0$), so the three models that we consider have this property.

Obviously geographical features (such as rivers and mountains) can introduce further effects, but the front speed is measured using data separated by very large distances (up to several thousand kilometers) and the high values of the correlation coefficients for the corresponding linear fits of times and distances (up to $r=0.85$ for the Khoi-khoi expansion of herders^18^ and $r=0.87$ for the southern Bantu expansion of farmers^19^) make it reasonable to develop models in homogeneous space. It is also worth to note that at present there are not enough data from independent observations to parametrize non-homogeneous models. In any case, here we are interested in the simplest possible physical models to answer the question that has motivated this study, namely whether non-isotropic dispersal can explain why the speed of some human range expansions is too fast to agree with demic models based on isotropic dispersal^18,19^.

### Model 1

A very simple model is to assume that individuals have probability $p$ to migrate forward (i.e., outwards from the invaded range), namely with ${\Delta }_{x}\ge 0$ or $-\pi /2\le \theta \le \pi /2$, and with probability $1-p$ to migrate backward (i.e., inwards to the invaded range), namely with ${\varDelta }_{x} < 0$ or $\theta \in$ (-$\pi ,-\pi /2){\cup }^{}(\pi /2,\pi )\,$. This simple assumption has been previously applied in microbiology, specifically to the spread of some brain tumors in which cell dispersal is biased outwards from the tumor core^29^. However, in that case the non-cohabitation Eq. (3) was used because the progeny of tumor cells can disperse away from their parent cells immediately after reproduction (i.e., there is no cohabitation effect). In contrast, here we have to use the cohabitation Eq. (5) because we are dealing with human populations^3,17,33,34^. As explained in the Introduction, the problem that we want to solve is that some front speeds are *faster* than those predicted by isotropic dispersal^18,19^. For this reason, obviously we have to consider the case $p > 0.5$. Model 1 is shown in Fig. 1 for $p=0.5$ (isotropic dispersal) and $p=1$ (which corresponds to the maximum possible dispersal anisotropy for this model). Using the normalization condition (10), we find that in model 1 the angular dispersal distribution is

11 $$\Phi (\theta )=\{\begin{array}{l}\frac{p}{\pi }\,if-\frac{\pi }{2}\le \theta \le \frac{\pi }{2}\\ \frac{(1-p)}{\pi }\,if\,\theta \in \left(-\pi ,\,-\frac{\pi }{2}\right){\cup }^{}\left(\frac{\pi }{2},\,\pi \right).\end{array}$$

Using this distribution and Eq. (8) in Eq. (6), we find that the front speed in model 1 is

12 $$c=\begin{array}{c}{\rm{\min }}\\ \lambda  > 0\end{array}\frac{aT+ln[{\sum }_{i=1}^{n}{p}_{i}(p[{I}_{0}(\lambda {r}_{i})+{L}_{0}(\lambda {r}_{i})]+(1-p)[{I}_{0}(\lambda {r}_{i})-{L}_{0}(\lambda {r}_{i})])]}{\lambda T},$$

where ${I}_{0}(\lambda {r}_{i})=\frac{1}{\pi }{\int }_{0}^{\pi }\exp [\lambda {r}_{i}\,\cos \,\theta ]d\theta$ is the modified Bessel function of the first kind and order zero, and ${L}_{0}(\lambda {r}_{i})=\frac{1}{\pi }({\int }_{0}^{\pi /2}\exp [\lambda {r}_{i}\,\cos \,\theta ]d\theta -{\int }_{\pi /2}^{\pi }\exp [\lambda {r}_{i}\,\cos \,\theta ]d\theta )$ is the modified Struve function of the fist kind and order zero. We have used Eqs. 3.339 and 8.551–2 in ref. ^40^. We have also assumed that the minimum possible speed is that selected by the front, a well-established result not only for differential^2^ but also for integro-difference^12^ equations. In the isotropic case ($p=1/2$) Eq. (12) reduces to Eq. (14) in^33^ or Eq. (9) in^17^, as it should.

**Figure 2:**
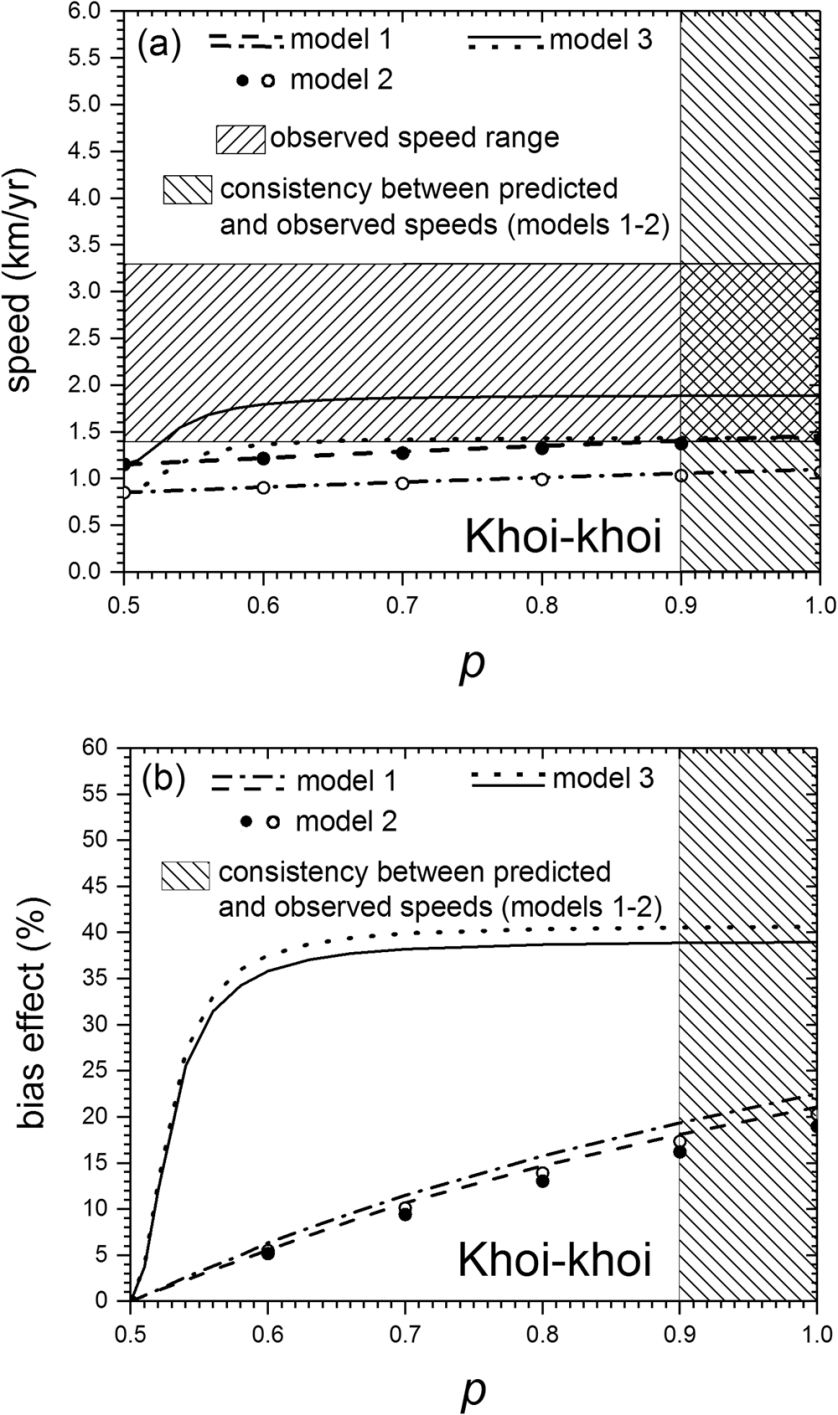
(**a**) Shows the speed of fronts for the Khoi-khoi expansion that transformed southwestern Africa from a land of hunter-gatherers into one of herders from 2,300 until 1,100 yr Before Present (BP). The observed range for the front speed, estimated from archaeological data, is shown as a horizontal hatched rectangle (1.4–3.3 km/yr)^18^. For each model, the range between the two curves is its predicted front speed. It is seen that models 1 and 2 are consistent with the observed front speed only if the latter is very close to its lower bound (1.4 km/yr). In contrast, model 3 is consistent with the observed front speed for a substantially wider range of the latter (up to 1.89 km/yr). This happens if $p\ge 0.53$ (this consistency range is not shaded for clarity), which implies that the bias does not need to be very strong (see Fig. 1 and the text below Eq. (17)). (**b**) Effect of the dispersal non-isotropy on the same Khoi-khoi front speed. For each value of the dispersal bias $p$, this effect is defined as the difference between the corresponding front speed minus the speed for non-biased dispersal ($p=0.5$), both of them obtained from (**a**), divided by the former and multiplied by 100. The effect is 18–23% for model 1 ($p\ge 0.9$), 16–20% for model 2 (same consistency range of $p$) and 17–41% for model 3 ($p\ge 0.53$).

In Fig. 2 we apply Eq. (12) to the spread of Khoi-khoi herders in Southwestern Africa (2,300-1,100 yr BP), for which the observed speed is 1.4–3.3 km/yr^18^ (horizontal hatched rectangle in Fig. 2a). This is a fast speed, if compared to that of the the spread of the most well-known case, namely the spread of the Neolithic in Europe (0.9–1.3^12^). The parameter values, already used in ref. ^18^, are $0.023\le a\le 0.033$ yr^−1^ for the initial growth rate (estimated archaeologically and ethnographically for several small preindustrial populations that settled into empty space), $29\le T\le 35$ yr (estimated from ethnographic data) and {*p*_*j*_}={0.67; 0.05; 0.04; 0.07; 0.08; 0.04; 0.05}, {*r*_*j*_}={0.5; 3; 7.5; 15; 25; 35; 95} km for the dispersal kernel (estimated for preindustrial herders^18^, see Supplementary Text S1 to the present paper). Using these parameter values into Eq. (12), we have obtained the dashed and dashed-dotted curves in Fig. 2a. The dashed curve corresponds to the maximum possible speed ($a=0.033$ yr^−1^ and $T=29$ yr) and the dashed-dotted curve corresponds to the minimum possible speed ($a=0.023$ yr^−1^ and $T=35$ yr). We see that model 1 (the range between the dashed and dashed-dotted curves) is consistent with the observed speed (horizontal hatched rectangle) but only for speeds close to the lower observed bound (1.4 km/yr) and sufficiently biased dispersal, namely $p\ge 0.9$. This range is shown as a vertical hatched rectangle in Fig. 2a,b. Figure 2b shows the effect of the dispersal bias, namely the speed $c$ from Eq. (12) minus the corresponding speed for non-biased dispersal ($p=1/2$), divided by the former and multiplied by 100. We see that according to model 1, consistency between the observed and predicted speeds (hatched vertical rectangle, i.e., $p\ge 0.9$) implies that the bias effect is between 18% and 23% (Fig. 2b).

**Figure 3:**
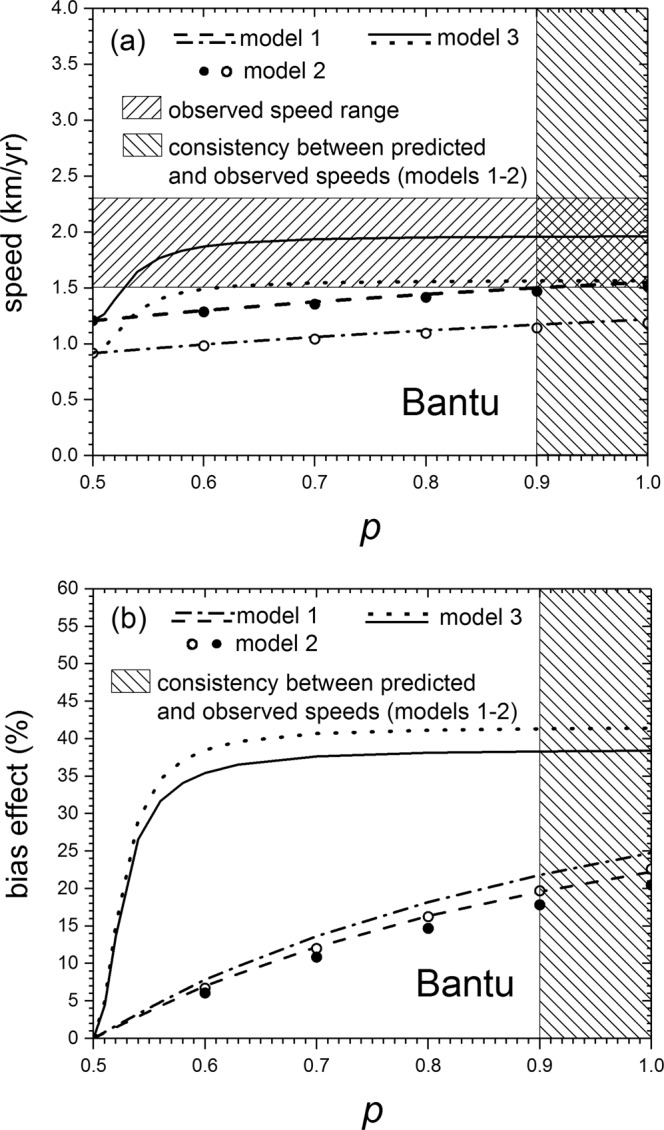
(**a**) Shows the speed of fronts for the Bantu expansion that transformed southeastern Africa from a land of hunter-gatherers into one of farmers from about 3,400 until 1,400 yr BP. The observed range for the front speed, estimated from archaeological data, is shown as a horizontal hatched rectangle (1.5–2.3 km/yr)^19^. For each model, the range between the two curves is its predicted front speed. In spite of the fact that the dispersal kernel is different from that used in Fig. 2 (because here we are dealing with farmers rather than herders), the conclusions are very similar. Indeed, models 1 and 2 agree with the observed front speed only if the latter is very close to its lower bound (1.5 km/yr in this case). In contrast, model 3 is consistent with the observed front speed for a substantially wider range of the latter (up to 1.96 km/yr), which happens if $p\ge 0.53$, so the bias does not need to be very strong (see Fig. 1 and the text below Eq. (17)). (**b**) The effect of the dispersal bias $p$ on the front speed of the same southern Bantu expansion is 19–25% for model 1 ($p\ge 0.9$), 18–23% for model 2 (same consistency range of $p$) and 19–42% for model 3 ($p\ge 0.53$).

In Fig. 3 we apply the same model 1, but to a different range expansion which was also rather fast, namely that of Bantu farmers in southeastern Africa (3,400-1,400 yr BP), for which the observed speed southwards from the Great Lakes area is 1.5–2.3 km/yr^19^ (hatched horizontal rectangle in Fig. 3a), using as in ref. ^19^ the same reproductive ranges as above ($0.023\le a\le 0.033$ yr^−1^, $29\le T\le 35$ yr) because farmers and herders have very similar reproductive dynamics^12,18^ and the dispersal kernel {*p*_*j*_}={0.40; 0.17; 0.17; 0.26}, {*r*_*j*_}={2.4; 14.5; 36.2; 60.4} km (estimated for preindustrial farmers^12,17,18^, see Supplementary Text S1). We see in Fig. 3a that model 1 is again viable, but only for $p\ge 0.9$ (vertical hatched rectangle) and sufficiently slow speeds. Interestingly, this is the same conclusion as for the Khoi-khoi expansion (Fig. 2a), in spite of the fact that the dispersal kernels are different. For the Bantu expansion the bias effect is 19–25% (Fig. 3b), very similar to the range 18–23% for the Khoi-khoi (Fig. 2b).

### Model 2

Model 1 is consistent with the Khoi-khoi and Bantu data, but only assuming that the speed was very close to its minimum possible value suggested by the archaeological data (Figs. 2a and 3a, dashed and dashed-dotted curves). It is thus reasonable to ask if a different non-isotropic model can agree with a wider range of the observed speed. Moreover, although model 1 is very useful conceptually, it has the obvious pitfall of assuming the same probability ($p$) for all forward directions ($-\pi /2\le \theta \le \pi /2$) and, similarly, the same probability ($1-p$) for all backward directions (see Eq. (11)). There is thus a discontinuity at $\theta =\pi /2$ (as seen in Fig. 1, curve for model 1 and $p=1$), which is a questionable feature intuitively. We introduce model 2 to avoid this limitation as follows,

13 $$\Phi (\theta )=\frac{1+(2p-1)\cos \,\theta }{2\pi }.$$

This model 2 has been used previously in human dispersal^34^ but only in the diffusive approximation (i.e., a diffusive coefficient or second-order moment instead of the complete dispersal kernel), and that approximation is nowadays known to be invalid for human populations^17^. As in model 1, the case $p=1/2$ corresponds to isotropic or non-biased dispersal (uniform distribution $\Phi (\theta )=1/(2\pi )$) and the case $p=1$ to the maximum possible bias allowed by this model (because we must have $\Phi (\theta )\ge 0$ for all values of $\theta$). Model 2 (Eq. (13)) is shown for $p=1/2$ and $p=1$ in Fig. 1. In this figure, comparison of model 2 to model 1 (both of them for $p=1$) shows that model 2 is much more acceptable intuitively, because the discontinuity is no longer present. We next determine how using model 2 instead of model 1 changes the conclusions concerning the compatibility of predicted and observed speeds for the Khoi-khoi and Bantu Neolithic expansions.

Using the distribution (13) and Eq. (8) in Eq. (6), we find that the front speed in model 2 is

14 $$c=\begin{array}{c}{\rm{\min }}\\ \lambda  > 0\end{array}\frac{aT+ln[{\sum }_{i=1}^{n}{p}_{i}(\,{I}_{0}(\lambda {r}_{i})+(2p-1){I}_{1}(\lambda {r}_{i}))]}{\lambda T},$$

where ${I}_{0}(\lambda {r}_{i})$ has been defined below Eq. (12), ${I}_{1}(\lambda {r}_{i})=\frac{1}{\pi }{\int }_{0}^{\pi }\exp [\lambda {r}_{i}\,\cos \,\theta ]\cos \,\theta \,d\theta$ is the modified Bessel function of the first kind and order one, and we have used Eqs. 3.339, 3.915–2, 8.406–3 and 8.447–2 in ref. ^40^.

Figure 2a,b include, for the same parameter values as for model 1 above, the results of model 2 (full and empty circles). It is seen that the speeds predicted by model 2 are very similar to those by model 1. Thus, interestingly, the discontinuity in model 1 does not necessarily imply that it cannot yield realistic results. According to Figs. 2b and 3b, the bias effect is 16–20% and 18–23%, respectively, i.e. slightly lower than for model 1.

### Model 3

Both models 1 and 2 yield predicted speeds similar to those observed only if the latter are close to their minimum possible values (lower side of the hatched horizontal rectangle in Fig. 2a,b). As mentioned at the beginning of the previous subsection, we are interested to know if there is some other biased-dispersal model that agrees with the observations for a wider range of the front speed. Intuitively, it is easy to note a drawback of both models 1 and 2, namely that they cannot describe populations for which the bias is arbitrarily large. This can be seen from Fig. 1, where for the maximum possible bias ($p=1$) we see that a large portion of individuals move in directions that are not close to that along which the front propagates ($\theta =0$), and for model 2 even towards the space that has been already invaded by other individuals ($\theta  < -\pi /2$ or $\theta  > \pi /2$). This is unlikely to describe all possible non-isotropic dispersal behaviors, because it is perfectly reasonable that individuals of an invading human population may migrate only in directions close to that of the invasion front ($\theta \approx 0$). For this reason, we introduce model 3 with a Gaussian dispersal distribution,

15 $$\Phi (\theta )=A\,{e}^{-{q}^{2}{\theta }^{2}},$$

where we have have used ${\theta }^{2}$ rather than $\theta$ in the exponent to ensure that $\Phi (-\theta )=$ $\Phi (\theta )$ (see the text below Eq. (10)). The normalization condition (10) implies that $A=\frac{q}{\sqrt{\pi }\,{\rm{erf}}(\pi q)}$, where ${\rm{erf}}(x)$ is the error function and we have used Eqs. 3.321,2 in ref. ^40^. In order to compare to models 1–2, it will be useful to introduce the following bias parameter

16 $$p=\frac{q+10}{20}.$$

As in models 1–2, the case $p=1/2$ (or $q=0$) corresponds to isotropic dispersal. Other forms of $p$ with this property are possible, but Eq. (16) has the advantage that we will be able to compare the speeds from models 1–3 in a single figure. Before doing so, note from Fig. 1 that the Gaussian distribution (15) (model 3) can describe situations in which many individuals move close to the front propagation direction ($\theta \approx 0$), whereas models 1–2 cannot. It is also seen that higher values of $p$ correspond to more strongly biased dispersal (i.e., a larger fraction of individuals migrating in directions close to $\theta =0$). This fraction can be arbitrarily large in model 3, and this corresponds to the fact that, in contrast to models 1–2, there is not any upper limit to the value of $p$ in model 3.

Using the distribution (15) and Eq. (8) in Eq. (6), we find that the front speed in model 3 is

17 $$c=\begin{array}{c}{\rm{\min }}\\ \lambda  > 0\end{array}\frac{aT+\,\mathrm{ln}[{\sum }_{i=1}^{n}{p}_{i}{\int }_{-\pi }^{\pi }d\theta \,A\,{e}^{-{q}^{2}{\theta }^{2}+\lambda {r}_{i}\cos \theta }]}{\lambda \,T}.$$

In contrast to models 1–2, it does not seem possible to solve this integral analytically. Therefore, we follow a different approach. For a given set of parameter values ($a$, $T$, {*p*_*j*_} and {*r*_*j*_}), we plot the quotient in the right-hand side of Eq. (17) and find its minimum numerically. In this way, we have computed the speeds for model 3 in Figs. 2a and 3a. We see that model 3 is consistent with the observed speed for a substantially wider range of the latter (up to about 1.9 km/yr) than models 1–2, and that this happens for $p > 0.53$, both for the Khoi-khoi expansion of herders (Fig. 2a) and the Bantu expansion of farmers (Fig. 3a), in spite of the fact that both populations have different dispersal kernels. Remarkably, $p=0.53$ is not an extremely isotropic case but, as shown in Fig. 1, it is intermediate between $p=0.5$ (non-biased case) and $p=0.55$ (which is mildly biased, in the sense that some individuals move even backwards, i.e. with $\theta  < -\pi /2$ or $\theta  > \pi /2$, in contrast to, e.g., model 3 with $p=1$ in Fig. 1). Thus we conclude that biased dispersal can explain the speed of both Neolithic fronts. According to model 3, the bias effect is 17–41% for the Khoi-khoi (using the consistency range above $p > 0.53$ in Fig. 2b) and 19–42% for the Bantu ($p > 0.53$ in Fig. 3b).

Finally we note that in the most extremely biased case (*p→*∞), the angular distribution (15) becomes a Dirac delta centered at $\theta =0$ and Eq. (17) reduces to the new, very simple result

18 $$c=\begin{array}{c}{\rm{\min }}\\ \lambda  > 0\end{array}\frac{aT+\,\mathrm{ln}[{\sum }_{i=1}^{n}{p}_{i}{e}^{\lambda {r}_{i}}]}{\lambda \,T},$$

which we have checked that agrees with the saturating speeds for model 3 and large values of $p$ in Figs. 2a and 3a, as it should.

## Conclusions

In this paper we have shown that when a population expands its range, the front speed can be substantially faster if the dispersal of individuals is biased towards the front propagation direction, which seems a very reasonable assumption because individuals will presumably tend to move towards places with more free space (lower population densities) than to places with less free space (higher population densities). Moreover, for two human expansions (one of herders and one of farmers) we have shown that this effect can explain the range of the front speed estimated from archaeological data.

The present paper has crucial implications on a very important issue in Archaeology, Anthropology and Genetics, namely the relative importance of demic and cultural diffusion. For the two case studies considered in this work (Figs. 2–3), it has been previously shown that cultural transmission (i.e., the conversion of hunter-gatherers into farmers)^12^ is a possible explanation of the fact that the observed front speeds are faster than those predicted by an isotropic, purely demic model ($p=0.5$ in Figs. 2–3)^18,19^. However, here we have shown that biased dispersal may also explain the speeds of both range expansions (Figs. 2–3, especially model 3). Thus understanding Neolithic front speeds is by no means trivial, in the sense that they have these two possible explanations (and perhaps many others, see Supplementary Texts S1 and S2). Here we have shown that at least two different explanations may agree with the observed data. We would like to stress, however, that both explanations may partly apply, with different relative importance depending on the front considered. The problem of estimating the relative importance of biased dispersal and cultural transmission may be solved in the future as follows. It is highly likely that in some years it will become possible to measure dispersal kernels of prehistoric individuals directly (not using ethnographic analogues as in this paper and all previous work), because ancient genetics has been already used to detect a few prehistoric parent-child pairs buried at different places^41,42^. When many such pairs are detected, it should become possible to determine the anisotropy in the dispersal kernel (e.g., parameter $p$ in our 3 models) in addition to the dispersal distances and probabilities (*r*_i_ and *p*_i_ in Eq. (8)). Then, the difference between the observed front speed and that predicted by a purely demic model with biased dispersal (e.g., models 1–3 in this paper) would be an estimation of the importance of cultural transmission (i.e., the conversion of hunter-gatherers into herders or farmers). This will hopefully make it possible to disentangle the roles of both complex mechanisms (cultural transmission on one hand, and biased dispersal on the other) in human range expansions.

The interest of our results is not limited to Neolithic inland spread, because there are also some data on Neolithic coastal^13,15^, Paleolithic^43^ and modern^8^ front speeds for which the dispersal bias effect could be of importance. Besides human range expansions, our approach can be applied to other social applications of statistical physics including innovation diffusion^22^, rumor propagation^23^, linguistic fronts^24^, diffusion in economic space^26^ and the evolution of cooperation in spatial systems^27^, because it seems reasonable to expect that such human systems may exhibit higher dispersal probabilities in directions closer to that of the front propagation (for the reason given at the beginning of this section). Our 3 models are easy to adapt to some other fields for which the cohabitation Eq. (5) must be replaced by the non-cohabitation one (3), including ecology (biological invasions^28^ and epidemic spread with biased dispersal due, e.g., to wind or water currents^25^), microbiology (growth of bacterial colonies and cancer tumors displaying biased dispersal^29^), medicine (virus treatment of tumors^30^), etc.

## Supplementary information

Supplementary Texts.

## Data Availability

No datasets were generated or analyzed during the current study.

## References

[1] Perc M (2019). The social physics collective. Sci. Rep..

[2] Fort J, Méndez V (2002). Wavefronts in time-delayed systems. Theory and comparison to experiment. Rep. Progr. Phys..

[3] Fort J, Pujol P (2008). Progress in front propagation research. Rep. Progr. Phys..

[4] Fisher RA (1937). The wave of advance of advantegeous genes. Ann. Eugen. (London).

[5] Kolmogorov AN, Petrovsky IG, Piskunov NS (1937). A study of the diffusion equation with increase in the amount of substance, and its application to a biological problem. Bull. Univ. Moscow, Ser. Int. A.

[6] Ammerman, A. J. & Cavalli-Sforza, L. L. A population model for the diffusion of early farming in Europe in *The explanation of culture change: models in prehistory* (ed. Renfrew, C.) 343-357 (Duckworth (1973).

[7] Ammerman, A. J. & Cavalli-Sforza, L. L. *The Neolithic transition and the genetics of populations in Europe* (Princeton University Press (1984).

[8] Bertuzzo E, Maritan A, Rodriguez-Iturbe I, Rinaldo A (2007). River networks and ecological corridors: reactive transport on fractals, migration fronts, hydrochory. Water Resources Res..

[9] Premack D, Premak A (2005). Evolution versus invention. Science.

[10] Reich, D. Who we are and how we got here. Ancient DNA and the new science of the human past (Pantheon Books (2018). 30047322

[11] Diamond J, Bellwood P (2003). Farmers and their languages: the first expansions. Science.

[12] Fort J (2012). Synthesis between demic and cultural diffusion in the Neolithic transition in Europe. Proc. Natl. Acad. Sci. USA.

[13] Isern N, Zilhao J, Fort J, Ammerman AJ (2017). Modeling the role of voyaging in the coastal spread of the Early Neolithic in the West Mediterranean. Proc. Natl. Acad. Sci. U.S..

[14] Davison K, Dolukhanov P, Sarson GR, Shukurov A (2006). The role of waterways in the spread of the Neolithic. J. Arch. Sci..

[15] Fort J (2003). Population expansion in the western Pacific (Austronesia): a wave of advance model. Antiquity.

[16] Fort J, Méndez V (1999). Time-delayed theory of the Neolithic transition in Europe. Phys. Rev. Lett..

[17] Isern N, Fort J, Pérez-Losada J (2008). Realistic dispersion kernels applied to cohabitation reaction-dispersion equations. J. Stat. Mechs. Theor. Exp..

[18] Jerardino A, Fort J, Isern N, Rondelli B (2014). Cultural diffusion was the main driving mechanism of the Neolithic transition in Southern Africa. PLos One.

[19] Isern N, Fort J (2019). Assessing the importance of cultural diffusion in the Bantu spread into southeastern Africa. PLoS One.

[20] Patterson MA, Sarson GR, Sarson HC, Shukurov A (2010). Modelling the Neolithic transition in a heterogenous environment. J. Arch. Sci..

[21] Fedotov S, Moss D, Campos D (2008). Stochastic model for population migration and the growth of human settlements during the Neolithic transition. Phys. Rev. E.

[22] Guardiola X, Díaz-Guilera A, Pérez JC, Arenas A, LLas M (2002). Modeling diffusion of innovations in a social network. Phys. Rev. E.

[23] Zhao H, Zhu L (2016). Dynamic Analysis of a Reaction–Diffusion Rumor Propagation Model. Int. J. Bifurcation & Chaos.

[24] Isern N, Fort J (2014). Language extinction and linguistic fronts. J. Roy. Soc. Interface.

[25] Belik V, Geisel T, Brockmann D (2011). Natural human mobility patterns and spatial spread of infectious diseases. Phys. Rev. X.

[26] Chatterjee R, Eliashberg J (1990). The innovation diffusion process in a heterogeneous population: a micromodeling approach. Management Science.

[27] Hauert C, Doebeli M (2004). Spatial structure often inhibits the evolution of cooperation in the snowdrift game. Nature.

[28] Shigesada, M. & Kawasaki, K. *Biological invasions: theory and practice* (Oxford University Press (1997).

[29] Fort J, Solé RV (2013). Accelerated tumor invasion under non-isotropic cell dispersal in glioblastomas. New J. Phys..

[30] de Rioja V, Isern N, Fort J (2016). A mathematical approach to virus therapy of glioblastomas. Biology Direct.

[31] Fort J, Jana D, Humet JM (2004). Multidelayed random walks: theory and application to the neolithic transition in Europe. Phys. Rev. E.

[32] Murray, J. D. *Mathematical biology* (Springer-Verlag (1993).

[33] Fort J, Pérez-Losada J, Isern N (2007). Fronts from integro-difference equations and persistence effects on the Neolithic transition. Phys. Rev. E.

[34] Fort J, Pujol T (2007). Time-delayed fronts from biased random walks. New J. Phys..

[35] Gillis J (1955). Correlated random walk. Math. Proc. Cambridge Philos. Soc..

[36] Kareiva PM, Shigesada N (1983). Analyzing insect movement as a correlated random walk. Oecologia.

[37] Codling EA, Plank MJ, Benhamou S (2008). Random walk models in biology. J. R. Soc. Interface.

[38] Shukurov A, Snodin AP, Seta A, Bushby PJ, Wood TS (2017). Cosmic rays in intermittent magnetic fields. Astrophys. J. Lett..

[39] Wadkin LE (2018). Correlated random walks of human embryonic stem cells *in vitro*. Phys. Biol..

[40] Gradshteyn, I. S. & Ryzhik, I. M. *Table of integrals, series, and products* (Academic Press (1994).

[41] Sánchez-Quinto F (2019). Megalithic tombs in western and northern Neolithic Europe were linked to a kindred society. Proc. Natl. Acad. Sci. U.S..

[42] Mittnik A (2019). Kinship-based social inequality in Bronze age Europe. Science.

[43] Fort J, Pujol T, Cavalli-Sforza LL (2004). Palaeolithic populations and waves of advance. Cambridge Archaeol. J..

