# Supplementary material for: Biased dispersal can explain fast human range expansions
Source: Sci Rep. 2020 Jun 3;10:9036. doi: 10.1038/s41598-020-66045-2 (PMC7271227; doi:10.1038/s41598-020-66045-2)
Supplement: Supplementary file 1 — Supplementary Texts. [file 41598_2020_66045_MOESM1_ESM.pdf]

Supplementary Texts

S1. Non-biased dispersal implies long distances

In this section we use a very simple, non-biased model and analyze to what extent the dispersal behavior of real preindustrial populations should be modified to obtain agreement with the observed spread rates (Khoi-khoi and Bantu) considered in the main paper.

It is very difficult to find real dispersal data per generation of preindustrial populations. Obviously this can depend on landscape, climate, the subsistence strategy, etc., but the data available at present are so scarce that only the role of the subsistence strategy has been discussed in some detail (it has been noted that the mobility of hunter-gatherers is higher than for farmers [1]). Table S1 includes the list of all preindustrial populations for which dispersal kernels per generation have been measured, to the best of our knowledge (the complete kernels are included in table S2). Hunter-gatherers are not included because the two expansions considered in the main paper deal with herders and farmers. Of course, it could be argued that some of these 11 rural populations are more genuinely preindustrial than others. But apparently their subsistence systems, means of transportation and other social features make it reasonable to classify all of them as preindustrial, not as industrialized populations. A recent work [2] has analyzed the differences between the dispersal kernels of a preindustrial population and an industrialized one, as well as the implications of those differences on the results of human spread models.

In table S1, population #1 is the only one of preindustrial herders for which the dispersal kernel is known (to the best of our knowledge). It has been applied in the main text to the range expansion of Khoi-khoi herders.

Populations # 2-4 in table S1 include a range (10-19 yr or 20-29 yr) which gives the ages of individuals for which the distance between their birthplace and place of residence was recorded. For African farmers (Bantu expansion), in the main text we have used the kernel of population # 3 (Gilishi 20-29yr) because the measured interval of the generation time (29-35 yr) is closer to the age interval of this population (20-29 yr) than to that of populations # 2 and # 4 (10-19 yr).

The Issongos (population #7 in table S1) are also African farmers but they have some special features: (i) a low mobility due to their very limited area and few marriages with other tribes [3], and (ii) a subsistence system not based on cereals [4] (see table S1), unlike the populations involved in the Bantu expansion southwards from the Great Lakes area [5]. For these reasons we have preferred not to use the kernel of the Issongos in the main paper. However, we think that it can be also useful for comparison purposes.

Column 5 in table S1 gives the maximum distance r_{max} in each measured kernel (see table S2 for the complete kernel). We focus on the role of r_{max} because it is well-known to have more influence on the spread rate than other kernel distances (this effect is called long-distance dispersal in Ecology [6]). In order to see if the non-biased model can explain fast human range expansions, we use Eq. (12) in the main paper with $p = \frac{1}{2}$ to obtain the results in column 6 in table S1 (this model is more precise than Fisher-type models [7]). Each value is the distance by

which r_{max} in the corresponding kernel should be replaced to obtain a maximum spread rate of 1.89 km/yr, similarly to model 3 in Fig. 3a in the main paper (Khoi-khoi expansion). The corresponding % in table S1 is this new, hypothetical value of r_{max} minus the original, real one (column 5) divided by the latter and multiplied by 100. The last two columns give the same results for the Bantu expansion (i.e., for 1.96 km/yr, see model 3 in Fig. 2a in the main paper).

#	population or area **(in bold if used in the main paper)**	main subsistence (H=herding F=farming G=gardening)	kernel from Refs.	r_{max} (km)	Khoi-khoi * r_{max} (km)	%	Bantu ** r_{max} (km)	%
**1**	**Kelardasht**	H (cattle, sheep) [8]	[8, 9]	95	158	66%	164	73%
2	Gilishi 10-19 yr	F (maize, sorghum) [10]	[11, 3, 7]	60.4	100.0	66%	103.7	72%
**3**	**Gilishi 20-29 yr**	F (maize, sorghum) [10]	[11, 3, 7]	60.4	97.5	61%	101.3	68%
4	Shiri 10-19 yr	F (maize, sorghum) [10]	[11, 3, 7]	60.4	78.1	29%	81.1	34%
5	Yanomamö M†	G (plantains) and F [12]	[13, 14, 7]	110	171	55%	181	65%
6	Yanomamö B††	G (plantains) and F [12]	[13, 2]	105††	171	63%	182	73%
7	Issongos	F (cassava, yams) [4]	[15, 7]	100	227	127%	236	136%
8	Markazi	F (rice) [8]	[8, 16]	97.65	212	117%	220	125%
9	Bihar	F (rice) [17]	[17, 16]	62.5	168	169%	175	180%
10	Varanasi	F (rice) [18]	[18, 16]	68	204	200%	213	213%
11	Dirang Monpa	F (maize) [19]	[19]	145	181	25%	189	30%

* distance by which r_{max} in the measured kernel (column 5 or table S2) should be replaced for the non-biased model to yield a maximum spread rate of 1.89 km/yr, similarly to model 3 in Fig. 3a in the main paper (Khoi-khoi expansion). The % is the new r_{max} minus original r_{max} divided by the latter and multiplied by 100.

** distance by which r_{max} in the measured kernel (column 5 or table S2) should be replaced for the non-biased model to yield a maximum spread rate of 1.96 km/yr, similarly to model 3 in Fig. 2a in the main paper (Bantu expansion). The % is the new r_{max} minus original r_{max} divided by the latter and multiplied by 100.

† Yanomamö M refers to mating distances, defined as distances between the birthplaces of mother and father.

†† Yanomamö B refers to distances between birthplaces of parent and child. We have used the histogram with 11 bins in Ref. [2].

Table S1. Details (columns 2-5) and results (using table S2) for preindustrial populations of herders and farmers the dispersal kernels of which have been measured in ethnographic fieldwork.

#	population or area **(in bold if used in the main paper)**	probabilities $\{p_1, p_2, p_3, \dots\}$	distances $\{r_1, r_2, r_3, \dots\}$ (km)
**1**	**Kelardasht**	$\{0.67, 0.05, 0.04, 0.07, 0.08, 0.04, 0.05\}$	$\{0.5, 3, 7.5, 15, 25, 35, 95\}$
2	Gilishi 10-19 yr	$\{0.54, 0.17, 0.04, 0.25\}$	$\{2.4, 14.5, 36.2, 60.4\}$
**3**	**Gilishi 20-29 yr**	$\{0.40, 0.17, 0.17, 0.26\}$	$\{2.4, 14.5, 36.2, 60.4\}$
4	Shiri 10-19 yr	$\{0.19, 0.07, 0.22, 0.52\}$	$\{2.4, 14.5, 36.2, 60.4\}$
5	Yanomamö M	$\{0.19, 0.54, 0.17, 0.04, 0.04, 0.02\}$	$\{10, 30, 50, 70, 90, 110\}$
6	Yanomamö B†	$\{5,13,33,22,33,19,20,5,5,3,2\}/160$	$\{5,15,25,35,45,55,65,75,85,95,105\}$
7	Issongos	$\{0.42, 0.23, 0.16, 0.08, 0.07, 0.02, 0.01, 0.01\}$	$\{2.3, 7.3, 15, 25, 35, 45, 55, 100\}$
8	Markazi	$\{0.803, 0.040, 0.022, 0.025, 0.063, 0.005, 0.009, 0.019, 0.014\}$	$\{0.5, 5.5, 15, 25, 35, 50.03, 57.20, 60.51, 97.65\}$
9	Bihar	$\{0.018, 0.081, 0.105, 0.129, 0.14, 0.125, 0.107, 0.079, 0.068, 0.057, 0.036, 0.025, 0.03\}$	$\{2.5, 7.5, 12.5, 17.5, 22.5, 27.5, 32.5, 37.5, 42.5, 47.5, 52.5, 57.5, 62.5\}$
10	Varanasi	$\{0.058, 0.122, 0.191, 0.256, 0.168, 0.101, 0.069, 0.023, 0.012\}$	$\{4, 12, 20, 28, 36, 44, 52, 60, 68\}$
11	Dirang Monpa	$\{171, 8, 10, 7, 1, 1, 0, 1, 1, 3, 4, 1, 1, 6\}/215$	$\{0,3,8,13,18,23,28,33,38,43,48,61,80,145\}$

†For the Yanomamö B we have used the histogram with 11 bins in Ref. [2].

Table S2. Dispersal kernels measured by ethnographic fieldwork for the preindustrial populations in table S1.

We note from table S1 that in order to explain the speed of the Khoi-khoi expansion (with a consistency range similar to model 3 in the main paper), the maximum distance r_{max} of the kernel used in the main paper (population #1 in tables S1 and S2) has to be increased substantially (by 66%). It can be argued that the necessary value of r_{max} (158 km) is not far from the maximum measured ones for preindustrial populations, which are 145 km and 110 km (populations # 11 and # 5 in table S1). This may suggest that perhaps biased dispersal and/or cultural transmission [20] are not really necessary to explain fast human range expansions. However, a kernel with a longer maximum distance does not necessarily lead to a faster front (because the speed depends also on the probability of r_{max} and on the other distances and probabilities). Thus, it is necessary to examine whether real kernels (especially those with the longest values of r_{max}, but also any other ethnographic kernels) can explain fast spread rates using the non-biased model. According to table S1 the kernels with highest values of r_{max} (populations # 11 and # 5) do not lead to speeds fast enough unless their values of r_{max} are also substantially increased (by 25% and 55%, respectively). Moreover, note that using the non-biased model none of all the 11 preindustrial populations for which dispersal kernels have been measured is able to explain (in a way similar to model 3 in our main paper) the fastness of neither the Khoi-khoi nor the Bantu expansions (because all percentages in table S1 are positive, i.e., the value of r_{max} has to be increased in all 22 cases). Our conclusion at this point is that the simplest model (non-biased dispersal without cultural transmission) is unable to explain fast human range expansions. We recognize, however, that this conclusion will be verified or falsified only when dispersal kernels of prehistoric populations are measured directly (as explained in the main paper, this may become possible in some years by using genetic methods).

S2. Alternative probability distributions

At first sight, it could seem that besides changing a characteristic dispersal distance (Sec. S1), it may be perhaps possible to develop non-biased models leading to fast spread rates in another way, namely by considering some widely-used probability distributions, e.g., Gaussian, exponential, log-normal, power-law, other Lévy flights, etc., for the dispersal kernel. Such distributions are used in many physical and microbiological applications because in those disciplines it is possible to measure characteristic dispersal distances for an enormous amount of particles (for example, in typical microbiological experiments there are 10^7-10^8 cells/ml [21]), so that their concentration as a function of space can be measured precisely and it often agrees precisely with one of the distributions mentioned. Similarly, for modern industrialized populations dispersal data can be recorded for a huge number (10^6-10^7) of distances and this makes it possible to determine the underlying distribution [22]. However, for pre-industrial populations we only have dispersal histograms (table S2) based on distance measurements per generation for a much smaller number of individuals (of the order 10-10^3). Such histograms they display irregular shapes that are probably more representative of the small number of data used than of an underlying probability distribution that can be safely inferred. For example, in table S2 the probability of dispersal for population #2 has its maximum at the smallest distance whereas that of population # 4 has it maximum at the longest distance, in spite of the fact that these are tribes of the same population (the Majangir [11]), they were observed during the same time interval, and they have the same subsistence system (table S1), environment, social customs, etc. Thus, in our opinion an important (and widely overlooked) point is that for human preindustrial populations it is highly questionable to fit a probability distribution with the data available at present, because this implies making an additional approximation. We think that it is preferable to use the original data (histograms in table S2) because in this way there is no need to assume the validity of any specific distribution. For example, it has been observed that fitting exponential distributions to measured kernels (such as those in table S2) in some cases widely overestimates the front speed (see Fig. 4 in Ref. [7]) as compared to using the measured data (dispersal histograms, such as those in table S2). This is why we have followed the latter approach (i.e., to use directly the recorded histograms rather than to fit a distribution). Just to summarize, we think that there is little point in fitting a distribution to human preindustrial dispersal kernels because (i) in some cases the approximation will be invalid (e.g., population C in Fig. 4 of Ref. [7]); or (ii) in other cases it will be valid, but the results will be very similar to those obtained by using the original histogram (e.g., population A in Fig. 4 of Ref. [7]), so fitting a distribution will not lead to a new result after all. Such a fit would be justified if the fitted distribution were a better description of reality that the measured data (to which the distribution itself is fitted), but it is difficult to believe this. As mentioned above, in many other applications there are millions of data, making it possible to construct histograms with thousands of bins, which are very difficult to handle and then it may be reasonable to fit a distribution.

References

[1] Fort J, Pujol T, Cavalli-Sforza LL, "Palaeolithic populations and waves of advance," *Cambridge Archaeol. J.,* vol. 14, pp. 53-61, 2004.

[2] Fort J, Pareta M M, "Long-distance dispersal and Neolithic waves of advance," *J. Arch. Sci.,* vol. 119, no. 105148, 2020.

[3] Ammerman A J, Cavalli-Sforza L L, The Neolithic transition and the genetics of populations in Europe, Princeton: Princeton Univeristy Press, 1984.

[4] Bahuchet S, Hladik C M, Hladik A, Dounias E, "Agricultural strategies as complementary activities to hunting and fishing," in *Food and nutrition in the African rain forest*, C. M. Hladik, S. Bahuchet and I. Garine, Eds., Paris, Unesco/MAB, 1990, pp. 31-35.

[5] Isern N, Fort J, "Assessing the importance of cultural diffusion in the Bantu spread into southeastern Africa," *PLoS One,* vol. 14, no. e0215573, 2019.

[6] Clark J S, «Why trees migrate so fast: confronting theory with dispersal biology and the paleorecord,» *Am. Nat.,* vol. 152, pp. 204-224, 1998.

[7] Isern N, Fort J, Pérez-Losada J, "Realistic dispersion kernels applied to cohabitation reaction-dispersion equations," *J. Stat. Mechs.: Theory and experiment,* vol. 2008, no. P10012, 2008.

[8] Mehrai H, A demographic study of some populations in Shahrestan Nowshahr, Mazandaran, Iran, with reference to the genetic structure (Ph. D. Thesis), Durham: Durham University, 1984.

[9] Jerardino A, Fort J, Isern N, Rondelli B, "Cultural diffusion was the main driving mechanism of the Neolithic transition in southern Africa," *PLoS One,* vol. 9, no. e113672, 2014.

[10] Stauder J, "Anarchy and ecology: political society among the Majangir," *J. Anthropol.,* vol. 28, pp. 153-168, 1972.

[11] Stauder J, The Majangir: ecology and society of a southwest Ethiopian people, Cambridge: Cambridge University Press, 1971.

[12] Chagnon N A, Yanomamö: the fierce people, 3rd. ed., New York: Holt, Rinehart and Winston, 1983.

[13] Biella P, Chagnon N A, Seaman G, Yanomamö interactive: the Ax fight (CD-ROM), Orlando: Harcourt Brace & company, 1997.

[14] MacDonald D H, Hewlett B S, "Reproductive interests and forager mobility," *Curr. Anthropol.,* vol. 40, pp. 501-524, 1999.

[15] Cavalli-Sforza L L, Bodmer W F, The genetics of human populations, New York: Dover, 1971.

[16] Cobo J M, Fort J, Isern N, "The spread of domesticated rice in eastern and southeastern Asia was mainly demic," *J. Arch. Sci.,* vol. 101, pp. 123-130, 2019.

[17] Singh N K, Singh B P, "Study of distance associated with marriage migration," *Int. J. Math. Comp. Appl. Res.,* vol. 5, pp. 111-116, 2015.

[18] Shukla K K, "Models to describe the distribution of distance associated with marriage migration," *J. Inst. Sci. Tech.,* vol. 20, pp. 145-147, 2015.

[19] Barua S, "Mating patterns among the Dirang Monpa of West Kameng district, Arunachal Pradesh," *Curr. Anthropol.,* vol. 27, pp. 188-190, 1986.

[20] Fort J., "Synthesis between demic and cultural diffusion in the Neolithic transition in Europe," *PNAS,* vol. 109, pp. 18669-18673, 2012.

[21] Fort J, Méndez V, "Time-delayed spread of viruses in growing plaques," *Phys. Rev. Lett.,* vol. 89, no. 178101, 2002.

[22] Zhao K, Musolesi M, Hui P, Tarkoma S, "Explaining the power-law distribution of human mobility through transportation modality decomposition," *Sci. Rep.,* vol. 5, no. 9136, 2015.
